# An Unusual Case of Pancreatic Ductal Adenocarcinoma With Synchronous Rectal Metastasis in a Younger Female

**DOI:** 10.1155/crgm/9940087

**Published:** 2026-06-14

**Authors:** Olanrewaju Adeniran, Farirai Marwizi, Abdullahi Sulaiman, Nolan Holley, Omotomilola Fadipe, Budoor Alqinai, Ayowumi Adekolu, Joshua Kirkpatrick, Ethan M. Cohen, Joseph Simmons, Mohamed Zitun, Asad Pervez

**Affiliations:** ^1^ Division of Internal Medicine, Department of Medicine, West Virginia University, Morganton, West Virginia, USA, wvu.edu; ^2^ Department of Internal Medicine, St. John’s Episcopal Hospital, Far Rockaway, West Virginia, USA, ehs.org; ^3^ Department of Pathology, Anatomy and Laboratory Medicine, West Virginia University, Morgantown, West Virginia, USA, wvu.edu; ^4^ Division of Gastroenterology and Hepatology, Department of Medicine, West Virginia University, Morgantown, West Virginia, USA, wvu.edu; ^5^ Division of Gastroenterology, Hepatology, and Nutrition, Allegheny Health Network, Pittsburgh, Pennsylvania, USA, ahn.org; ^6^ Community Howard Regional Hospital, Kokomo, Indiana, USA; ^7^ Virginia Mason Franciscan Health System, Seattle, West Virginia, USA

**Keywords:** immunohistochemistry, pancreatic ductal adenocarcinoma (PDAC), synchronous rectal metastasis, young women

## Abstract

**Background:**

Pancreatic cancer (PC) remains one of the leading causes of cancer mortality in the United States. There has been a concerning trend in recent years where younger females are experiencing a faster annual percentage increase than their male counterparts (2.36% vs. 0.62%). Metastasis of pancreatic ductal adenocarcinoma (PDAC) to the rectum is uncommon, and synchronous rectal metastasis occurs even less frequently.

**Case Presentation:**

We describe a case of a 49‐year‐old woman with Stage IV PDAC with synchronous rectal metastases. The hallmark of her symptoms was hematochezia and epigastric pain, initially misdiagnosed as gastritis. However, the patient returned with new laboratory and imaging findings of the synchronous pancreatic tail and rectal masses confirmed by histology. Immunohistochemistry was crucial in distinguishing a primary PC from a rectal primary. The patient continues to undergo chemotherapy 14 months after presentation.

**Conclusion:**

This represents the first documented case in the United States and the sixth reported case of synchronous rectal metastasis from PDAC. It highlights the increasing incidence and the demographic shift of PC among young women in the United States, which needs further investigation. The case adds to our knowledge of the unusual metastatic pattern of PDAC and the need for a multidisciplinary approach, including the role of immunohistochemistry in distinguishing between the primary etiology of malignancy to establish an accurate diagnosis, instituting an appropriate treatment plan, and preventing unwanted treatment and surgeries.

## 1. Introduction

Pancreatic cancer (PC) is now the third leading cause of cancer‐related mortality in the United States (US), with an estimated 5‐year survival rate of about 12.8% [1]. Given the deep location of the pancreas in the abdomen, the lack of screening guidelines for the general population, and the nonspecificity of symptoms, more than 50% of patients with PC present with Stage IV disease [2]. The median age at diagnosis is about 71 years, with the incidence increasing significantly with age: 99.5 per 100,000 in those ≥ 80 and 1 per 100,000 in the 30–39 age group [3]. Although males have consistently been diagnosed at a higher incidence rate than females across all age groups and ethnicities, 14.8 vs. 11.6 per 100,000, recent studies have shown an increase in the average annual percentage change (AAPC) among younger women (55) than their male counterparts (2.36% vs. 0.62%) [4–6].

Pancreatic ductal adenocarcinoma (PDAC), the most common form of PC, accounts for over 90% of all pancreatic malignancies worldwide [7]. Metastatic PDAC typically carries a poor prognosis with a median survival rate of 8.5 to 11.1 months with current treatment modalities [8]. These metastases can be synchronous (within 6 months of the primary cancer) or metachronous (after 6 months of the primary cancer), with the most common sites of metastasis being the liver, bile duct, stomach, duodenum, and peritoneum. Although the actual prevalence is unknown, PDAC with synchronous metastasis is uncommon, and synchronous rectal involvement is even less frequent [9].

We present the first reported case of PDAC with synchronous rectal metastasis in the US and only the sixth case globally. We also emphasize the crucial role of immunohistochemistry in distinguishing between primary and metastatic disease. In addition, we provide further insight into the rising incidence rate of PC among younger women in the US.

## 2. Case Presentation

A 49‐year‐old female with a history of gastroesophageal reflux disease (GERD) presented to the emergency department (ED) with 3 months of persistent epigastric pain, hematochezia, fatigue, and a reported 6‐pound weight loss. She denied tobacco or alcohol use, any significant family history of pancreatic disease, or known genetic predispositions. The patient noted that three months before this encounter, she presented to an outside ED with mild epigastric pain for which multiple benign‐appearing cysts were identified in her liver and pancreatic head on a computed tomography scan (CT scan) and subsequent magnetic resonance imaging (MRI) (Figure 1). She was advised that her symptoms were gastritis‐related and discharged home on a proton pump inhibitor.

**FIGURE 1:**
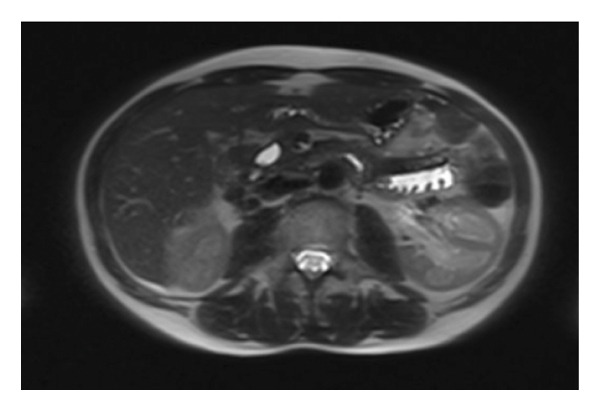
MRI of the abdomen and pelvis obtained outside the hospital, notable for a well‐circumscribed T2 hyperintense mass at the head of the pancreas measuring 1.8 × 1.0 cm in greatest axial dimensions without significant enhancement. No associated enhancing nodularity was seen. The main pancreatic duct demonstrates normal caliber.

This time, the patient was mildly tachycardic but otherwise hemodynamically stable. Abdominal exam was unremarkable. Workup revealed a deranged liver panel with alanine aminotransferase 439 U/L, aspartate aminotransferase 144 U/L, total bilirubin 1.7 mg/dL, and lipase 3150 U/L. Serum testing for cancer antigen 19–9 (CA 19–9) was significantly elevated, initially at 18,919 and peaking at 94,653 just before chemotherapy initiation. A new MRI study showed similar pancreatic head and hepatic cysts, as well as a new 22‐mm irregular hypoechoic pancreatic tail mass encasing the splenic vein, ill‐defined hepatic hilar masses with near‐complete portal venous occlusion, and dilated intra‐ and extrahepatic bile ducts. There is an abnormal, near‐circumferential wall thickening of the rectum with a mid‐rectal mass measuring at least 45 mm in craniocaudal dimension, with an ill‐defined adjacent soft tissue plane between the rectum and the external cervix and uterus. No definite adenopathy was noted in the pelvis (Figures 2 and 3). Trace ascites was noted in the pelvis.

**FIGURE 2:**
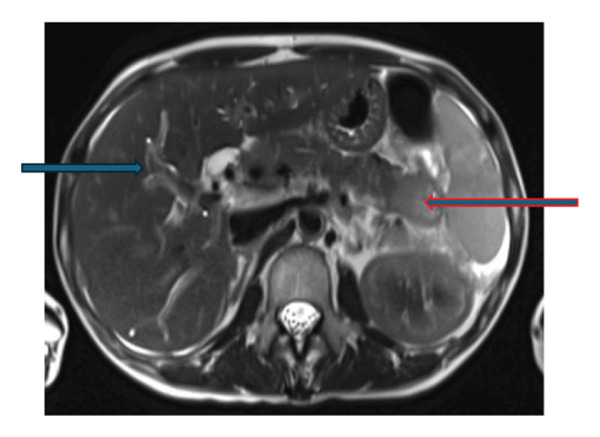
MRI at the time of presentation shows an ill‐defined hilar soft tissue mass with abnormal upstream intrahepatic biliary dilatation (blue arrow). Redemonstration of cystic lesions in the pancreatic head without pancreatic ductal dilatation or suspicious features. New ill‐defined soft tissue mass with necrosis in the pancreatic tail measuring 29 × 24 cm (red arrow). The splenic vein appears occluded.

**FIGURE 3:**
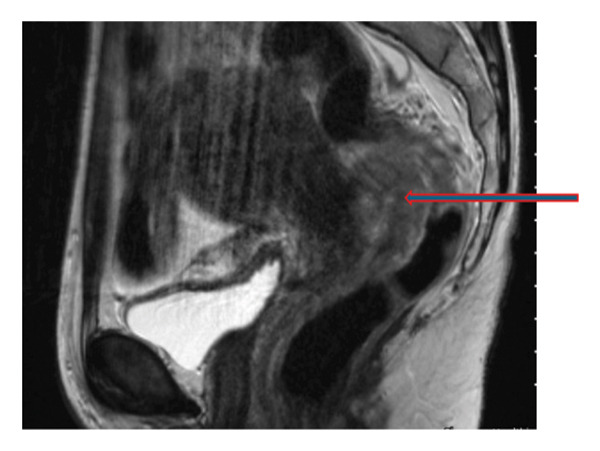
MRI of the pelvis at the time of presentation demonstrated the presence of a mid‐rectal mass measuring at least 45 mm in craniocaudal dimension (red arrow).

The patient subsequently underwent endoscopic ultrasound (EUS) and endoscopic retrograde cholangiopancreatography (ERCP), revealing additional obstructive masses within the right and left hepatic ducts, which were stented to ensure duct patency. Flexible sigmoidoscopy then confirmed a malignant appearing, partially circumferential, infiltrating rectal mass located around 10 cm above the anus (Figure 4). Histopathology of the pancreatic tail revealed histomorphological features similar to those of the rectum, both of which were concerning for malignancy: cellular atypia, a high nuclear‐to‐cytoplasmic ratio, and nuclear pleomorphism (Figures 5a and b). These similarities are further supported by their common immunohistochemical staining: both tissues stained positively for pancreatic markers such as Hepatocyte nuclear factor‐1beta (HNF1Beta), Cytokeratin (CK) 7, and retained (positive) mother against Decapentaplegic homolog 4 (SMAD4), which further demonstrates similar prognostic implications. Both tissues were also negative for colonic markers, including CK20, Caudal type home box 2 (CDX2), and Special AT‐rich sequence‐binding protein 2 (SATB2) (Figures 6a and b) [10]. This identical IHC profile raised suspicion for metastatic pancreatic adenocarcinoma. Histopathology of the porta hepatis revealed reactive inflammatory cells.

**FIGURE 4:**
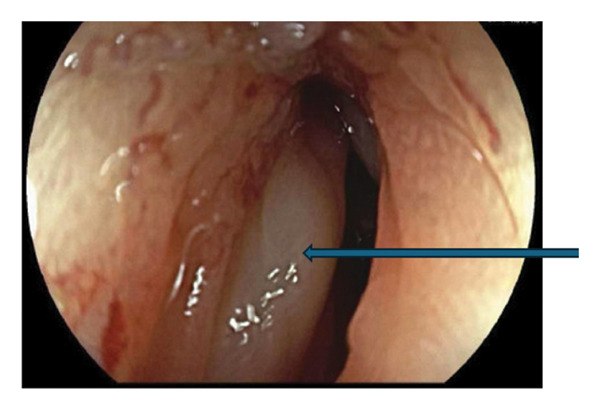
Flexible sigmoidoscopy while on admission, notable for malignant, partially obstructing tumor in the rectum starting at 10 cm proximal to the anus (blue arrow).

**FIGURE 5:**
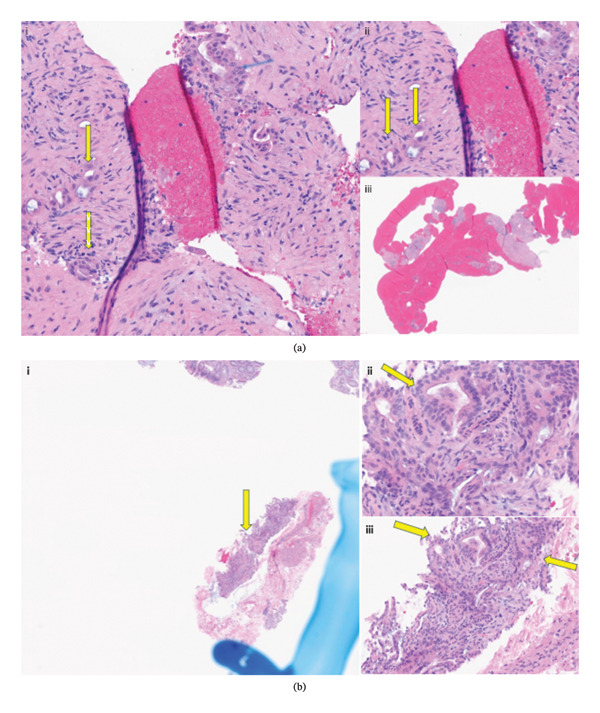
(a) Top: hematoxylin and eosin (H&E) staining of the pancreas showing biopsy portion of the pancreas tissue at magnifications × 400 (i), × 200 (ii), and × 20 (iii), showing malignant cells (arrows) with hyperchromatic nuclei, nuclear pleomorphism, stromal invasion, and background stromal reaction. (b) Bottom: H&E staining of the rectal mucosa showing photomicrographs at × 20 (i), × 200 (ii), and × 400 (iii), respectively. i. A low‐power view of normal rectal mucosa with a focus on the rectal lesion (arrow). ii. Malignant cells with a high nuclear‐to‐cytoplasmic ratio and atypia (arrow). iii. A high‐power view of malignant glands.

**FIGURE 6:**
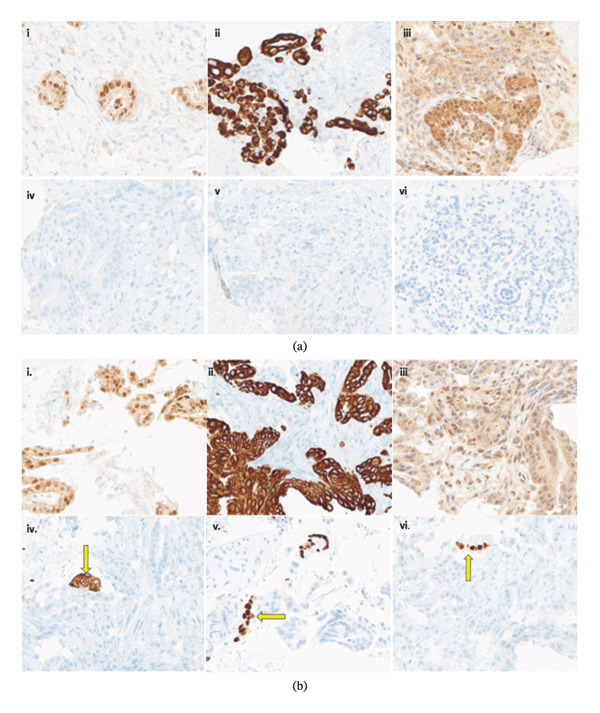
(a) Top: immunohistochemical staining of the pancreas. i‐iii are positive markers supporting a pancreatic origin: i. shows HNF1Beta in malignant cells, ii. shows cytokeratin 7 positivity, and iii. shows retained SMAD4, all supporting the diagnosis of pancreatic adenocarcinoma. iv‐vi are negative colonic markers: iv. shows cytokeratin 20 (KRT 20) negativity, v. showing CDX2 negativity, and vi. showing SATB2 negativity. These findings make metastasis from colorectal origin unlikely. All photomicrographs are at × 400 magnification. (b) Bottom: immunohistochemical staining of the rectum. i‐iii. are positive pancreatic markers: i. shows HNF1Beta in malignant cells, ii. shows cytokeratin 7 positivity, and iii. shows retained SMAD4, all supporting possible metastasis of pancreatic adenocarcinoma to the rectum. iv‐vi are negative colonic markers within the rectal tissue. iv. shows cytokeratin 20 negativity, v. shows CDX2 negativity, and vi shows SATB2 negativity. Arrows showing positivity of HNF1 Beta, cytokeratin, and SMAD4 in normal colonic mucosa (positive internal control), which further supports a negative rectal primary. All photomicrographs are at × 400 magnification.

A follow‐up positron emission tomography/CT (PET/CT) showed hypermetabolic areas in the pancreatic tail, porta hepatis, rectum, and pararectal tissues (Figures 7a and b). There was no activity along the main portal vein, which correlates with the reactive inflammatory cells reported on pathology and indicates it is not a metastatic site. With this, the patient was determined to have Stage IV PDAC with rectal metastasis and was started on FOLFIRINOX for both the pancreatic and rectal cancer. The patient has completed 10 cycles since initiation. Chemotherapy was generally tolerated, though from the 5th to 6th cycle, therapy was complicated by nausea, vomiting, and significant weight loss. She lost about 35 lbs while on chemotherapy and now weighs 67 lb. A few cycles had to be terminated to optimize nutrition and electrolyte levels. 24 months in, the patient has opted for hospice care following extensive goals‐of‐care discussions.

**FIGURE 7:**
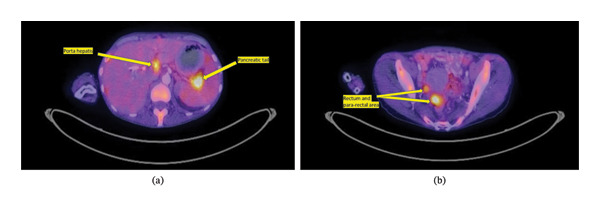
(a) Axial PET‐CT image showing intense focal FDG uptake in the pancreatic tail, corresponding to the primary pancreatic lesion, with additional mild hypermetabolic activity at the porta hepatis, consistent with reactive inflammatory changes (yellow arrows). (b) Axial PET‐CT image demonstrating focal hypermetabolic activity within the rectum and adjacent pararectal region, corresponding to areas of increased fluorodeoxyglucose (FDG) uptake (yellow arrows).

## 3. Discussion

We present a case of PDAC with synchronous rectal metastasis in a middle‐aged female. While the reasoning behind the rising AAPC of PC among young and middle‐aged women is not yet fully understood, the increased risk is particularly notable among African American women and in those with PDAC, suggesting a strong role for racial demographics and tumor characteristics in this trend [11]. Other associated factors include the changing prevalence of lifestyle risk factors such as smoking, obesity, and Type 2 diabetes among younger women [4]. Despite this rising incidence, younger women have a better overall survival compared to their male counterparts, attributable to their higher likelihood of receiving more aggressive treatments such as chemotherapy and/or surgery [12]. This resonates with our patient, who underwent 10 sessions of chemotherapy and has survived for 24 months since chemotherapy was initiated for Stage IV PDAC, although she has now elected hospice care due to declining functional status and cumulative treatment‐related toxicities she began developing around the 5^th^ cycle.

PDAC is known for its propensity to spread rapidly, both locally and hematogenously. Studies have suggested that this early dissemination can develop independently and in parallel to the primary tumor, even in those with localized disease [13]. According to the American College of Radiology (ACR), localized PDAC has a better 5‐year survival rate than metastatic disease (26% vs. 2%) [14]. Given these differences, there is strong justification for adopting a less stringent screening approach to facilitate earlier detection and improve patient outcomes by screening more high‐risk individuals with specific genetic and familial risk factors or with chronic abdominal symptoms of unclear etiology, as in our case [15].

PDAC with metastasis, particularly synchronous metastasis to the rectum, is unusual [9]. The mechanism of rectal spread is believed to occur through direct seeding of tumor cells into the serosa after breaching the pancreatic capsule, given that both the pancreas and the distal rectum are extraperitoneal [16]. Diagnosing PDACs typically involves imaging, endoscopy, and histology, as the sensitivity of imaging alone varies by technique. Specifically, CT scans have a sensitivity of 85%–95%, like MRI scans, but unlike MRIs, they are less sensitive for detecting tumors less than 2 cm [17, 18]. PET/CT demonstrates the most sensitive imaging modality for detecting primary PDAC and metastatic disease (100%) [19]. EUS is an important diagnostic test for PDAC, as it has a 99% sensitivity (99%) and is effective for evaluating local vascular invasion and obtaining tissue samples for histological diagnosis [17]. Staging laparoscopy is often recommended to detect occult peritoneal carcinomatosis that is missed on imaging [20]. This could be useful, particularly for pancreatic tail masses, which tend to implant in the adjacent peritoneum, especially the rectouterine pouch of Douglas. Although our clinical findings and imaging support hematogenous or lymphatic metastasis, the absence of open surgical exploration or staging laparoscopy precludes definitive exclusion of peritoneal implantation in the rectouterine pouch of Douglas from the pancreatic tail, with secondary rectal wall invasion. These procedures were not performed as they were unlikely to change the patient’s palliative management plan after diagnosis. While serum CA19‐9 is a commonly used biomarker for PC, its diagnostic utility is limited by its poor sensitivity and specificity [21]. Despite these diagnostic modalities, immunohistochemical staining of tissue samples is necessary to differentiate primary from metastatic disease, which is key to determining appropriate treatment modalities and avoiding the institution of the wrong treatment regimens or surgeries [22, 23].

Although our patient was deemed unsuitable for surgery due to Stage IV disease with porta hepatis involvement causing near‐complete portal venous occlusion, the potential for resection in oligometastatic PDAC remains an evolving area of study. In selected patients with limited metastatic burden, favorable performance status, and a durable response to systemic chemotherapy, surgery after FOLFIRINOX has been investigated, particularly when metastatic sites are solitary and amenable to surgical resection [8, 9]. Nevertheless, the available evidence remains limited, and there are no common guidelines recommending routine resection for metastatic PDAC, except in specific, well‐selected situations. In our case, despite prolonged survival and partial disease control with chemotherapy, the extent of local regional vascular involvement, biliary obstruction, severe treatment‐related cachexia, and declining functional status made it unlikely that a curative surgical resection would confer any significant advantage.

Differentiating metastatic PDAC remains challenging. Despite the lack of specific biomarkers, immunohistochemistry is often needed to distinguish between primary and secondary metastasis. HNF‐1 B, an important transcription factor for pancreatic development, is expressed in about 84.3% of PDACs and was positive in the patient’s pancreatic tissue but negative in the rectal mucosa, convincingly highlighting the mass to be consistent with a metastatic spread from the pancreas rather than a rectal primary [24, 25]. The pancreatic tissue also demonstrated a strong positive CK7 pattern, confirming the epithelial origin of the cells and favoring PDAC [10].

In addition to a negative CK20, other biomarkers used to identify carcinomas of colorectal origin, such as SATB2 and CDX2, were negative in the rectal specimen, further supporting the diagnosis of PDAC (Figures 5 and 6). Both tissues retained the SMAD4 gene. SMAD4, a tumor suppressor gene that regulates cell growth and differentiation, is inactivated (negative staining) in approximately 50%–55% of PDAC cases and retained (positive staining) in up to 50% [26]. Loss of the SMAD4 gene has been shown to increase the risk of PC progression and metastasis [26]. Its significance lies in its prognostic implications: patients with PDAC that express the SMAD4 gene tend to have longer survival than those with SMAD4 loss (median survival 19.2 vs. 14.7 months after resection) [26]. Ongoing multicenter studies have also linked SMAD4 alterations to increased metastatic progression in patients receiving FOLFIRINOX [27]. The preserved SMAD4 expression in both pancreatic and rectal samples may further explain our patient’s prolonged response to FOLFIRINOX chemotherapy, despite the median survival of 8.5–11 months. Given the absence of any clinical symptoms (e.g., dyspnea, tachypnea, and decreased oxygen saturation), radiologic (on CT and PET/CT) or pathologic features suggesting pulmonary involvement, a Thyroid transcription factor‐1 (TTF‐1) immunostaining was deemed not necessary.

At the time of this report, only 10 cases of PC with rectal metastasis had been described in the literature, all of which were reported outside the US (Table 1). Among the 10 cases, two were females, and only one was below 40. Five of the 10 cases demonstrated a synchronous metastatic association. Of these five, one was initially misdiagnosed as a primary rectal malignancy, leading to a postoperative adjustment in the chemotherapeutic regimen [22]. This underscores the importance of adequately differentiating between pancreatic and rectal cancers. In addition, three of the cases had no prior imaging to rule out a possible metachronous association. In one instance, colorectal metastasis from PDAC was detected only after combined colorectal and pancreatic resection [23, 28]. These interventions could have been avoided if the immunohistochemical diagnosis had been known earlier.

**TABLE 1:** Summary of previously reported primary pancreatic cancers with rectal metastasis.

Author/reported years.	Age	Gender	Clinical history	Association	IHC	Regimen	In contrast with our study.
Ohara et al. (2018)	67	Male	After 24 and 28 months of PACC resection, the patient developed liver and rectum metastases	Metachronous	Pancreatic and rectal tissues:CK‐7: +CK‐19: +CK‐20: âˆ’CDX2: âˆ’	Adjuvant chemotherapy for 11 months after PACC resection	Metachronous spread, Japanese ssudy
Fukatsu et al. (2009)	87	Male	Colonoscopy showed mucosal fold convergence in the rectum, while the CT scan showed a tumor in the pancreas and multiple metastatic masses in the liver and omentum	Synchronous	Biopsy specimens from rectum:CK‐7: +CK‐20: PSA neg	Best supportive care	No recent imaging authenticates a synchronous association, Japanese study
Sun et al. (2020)	75	Male	Developed rectal cancer 2 years after resection of primary pancreatic cancer	Metachronous	Rectal staining: CK7(+)], CK19(+)], CDX2(+)], Ki‐67(+), MUC1(+)] and negative for cytokeratin 20 [CK20(−)] and MUC2(−)	NA	Metachronous, head of the pancreas, Chinese study
Ejtehadi et al. (2014	79	Female	Anorectal Ca 4 week after Whipple’s procedure for ampullary adenocarcinoma with no initial evidence of metastasis	Metachronous	Pancreas: CK7/CK17/MUC1) positive and (CK20/CDX2/MUC2) negative Same for rectal stain	NA	Metachronous, pancreatic ampulla. United Kingdom study
O′Sullivan et al. (2022)	82	Male	PDAC with rectal metastasis	Synchronous	CK7+ mucosal CK20+ but negative submucosal CK20 immunostaining	N/A	Rectal primary, New Zealand study
Tianzhi et al. (2022)	43	Male	PDAC with rectal metastasis, initially treated as rectal cancer	Synchronous	Pancreas: strongly positive for CK 7 (A) but negative for CK‐20 (B) and CDX2 (C)	Oxaliplatin with capecitabine, switched to paclitaxel–albumin and gemcitabine	Diagnosis made postoperatively via requiring a change in the chemotherapy regimen, Chinese study
Chen et al. (2023)	71	Male	Rectal cancer 9 months after resection of pancreatic cancer	Metachronous	CK 7+, CDX2 + Ki‐67 +, but CK 20 and Mucoprotein 2 were negative	Lap pancreatectomy + splenectomy + gemcitabine and capecitabine. Paclitaxel for injection (albumin‐bound), TS‐1, and nimotuzumab for rectal cancer	Metachronous, a Chinese study
McKenzie et al. (2015)	55	Male	Rectal mass 2 years after pancreatic resection	Metachronous	Rectal mass: strongly + CK 7, negative cancer antigen 19–9, and no mucin	Surgical resection	Metachronous, Australian study
Giri et al. (2023)	60	Male	Pancreatic head cancer presents with jaundice and intestinal obstruction from rectal stenosis	Synchronous	CK +7 and CK+19, suggesting a pancreatic origin	NA	The pancreas head has no recent imaging that authenticates a synchronous association, an Indian study
Carrara et al. (2018)	38	Female	Mucinous cystic neoplasm of the pancreas with rectal metastasis	Synchronous	CK7+, CK20+, CDX2−/+),	NA	No recent imaging authenticates a synchronous association, Italian study

*Note:* CK, cytokeratin; CDX2, Caudal type home box 2; SATB2, Special AT‐rich sequence‐binding protein 2; MUC, mucoprotein; PDAC, pancreatic ductal adenocarcinoma; TS‐1, Tegafur‐gimeracil‐oteracil‐1.

Abbreviations: CT, computed tomography; PACC, pancreatic acinar cell carcinoma; PSA, prostate‐specific antigen.

We present the first case of PDAC with synchronous metastasis in the US and the sixth case worldwide. Our case is unique, as it is not only the first documented case in the US but also offers insight into the surging trend of PDAC among younger women. Making the distinction between primary pancreatic versus primary rectal cancer is paramount to limiting the harm to patients caused by erroneous treatment modalities, including surgeries. A multidisciplinary approach and early diagnosis through immunohistochemistry are crucial to note this difference.

## Author Contributions

Olanrewaju Adeniran drafted the case report and helped with draft revision. Farirai Marwizi, Joshua Kirkpatrick, Nolan Holley, Budoor Alqinai, and Omotomilola Fadipe helped with draft preparation and draft revision. Abdullahi Sulaiman assisted with histological interpretation and preparation. Ayowumi Adekolu, Ethan M. Cohen, Joseph Simmons, Mohamed Zitun, and Abdullahi Sulaiman helped conceive and design the manuscript and reviewed the manuscript critically for important intellectual content and grammatical edits.

## Funding

This study was not supported by any sponsor or funder.

## Disclosure

All authors have read and agreed to the published version of the manuscript.

## Ethics Statement

In accordance with the West Virginia Network of Ethics Committee, ethical approval is not required for this retrospective review of patient data.

## Consent

The patients gave written informed consent for the publication of this case report and any accompanying images.

## Conflicts of Interest

The authors declare no conflicts of interest.

## Data Availability

All the data used to support the findings of this case series are available as part of the article and references. Further inquiries can be directed to the corresponding author.
